# Distinct Predictive Immunogenomic Profiles of Response to Immune Checkpoint Inhibitors and IL2: A Real-world Evidence Study of Patients with Advanced Renal Cancer

**DOI:** 10.1158/2767-9764.CRC-21-0153

**Published:** 2022-08-30

**Authors:** Joel R. Eisner, Kirk D. Beebe, Gregory M. Mayhew, Yoichiro Shibata, Yuelong Guo, Carol Farhangfar, Farhang Farhangfar, Joshua M. Uronis, Jill Mooney, Michael V. Milburn, David Foureau, Richard L. White, Asim Amin, Marcos E. Milla

**Affiliations:** 1 GeneCentric Therapeutics, Inc., Durham, North Carolina.; 2 Levine Cancer Institute, Atrium Health, Charlotte, North Carolina.; 3 Synthorx, Inc – A Sanofi Company, La Jolla, California.

## Abstract

Recombinant human high-dose IL2 (HD-IL2; aldesleukin) was one of the first approved immune-oncology agents based upon clinical activity in renal cell carcinoma (RCC) and metastatic melanoma but use was limited due to severe toxicity. Next-generation IL2 agents designed to improve tolerability are in development, increasing the need for future identification of genomic markers of clinical benefit and/or clinical response. In this retrospective study, we report clinical and tumor molecular profiling from patients with metastatic RCC (mRCC) treated with HD-IL2 and compare findings with patients with RCC treated with anti-PD-1 therapy. Genomic characteristics common and unique to IL2 and/or anti-PD-1 therapy response are presented, with insight into rational combination strategies for these agents.

Residual pretreatment formalin-fixed paraffin embedded tumor samples from *n* = 36 patients with HD-IL2 mRCC underwent RNA-sequencing and corresponding clinical data were collected. A *de novo* 40-gene nearest centroid IL2 treatment response classifier and individual gene and/or immune marker signature differences were correlated to clinical response and placed into context with a separate dataset of *n* = 35 patients with anti-PD-1 mRCC.

Immune signatures and genes, comprising suppressor and effector cells, were increased in patients with HD-IL2 clinical benefit. The 40-gene response classifier was also highly enriched for immune genes. While several effector immune signatures and genes were common between IL2 and anti-PD-1 treated patients, multiple inflammatory and/or immunosuppressive genes, previously reported to predict poor response to anti-PD-L1 immunotherapy, were only increased in IL2–responsive tumors.

These findings suggest that common and distinct immune-related response markers for IL2 and anti-PD-1 therapy may help guide their use, either alone or in combination.

**Significance:**

Next-generation IL2 agents, designed for improved tolerability over traditional HD-IL2 (aldesleukin), are in clinical development. Retrospective molecular tumor profiling of patients treated with HD-IL2 or anti-PD-1 therapy provides insights into genomic characteristics of therapy response. This study revealed common and distinct immune-related predictive response markers for IL2 and anti-PD-1 therapy which may play a role in therapy guidance, and rational combination strategies for these agents.

## Introduction

IL2 is an essential cytokine for proliferation and survival of T and natural killer (NK) cells. Soon after its discovery in 1976 (1), immuno-oncologists recognized the potential for IL2 as a cancer therapy given its ability to generate “lymphokine-activated killer cells”, later identified as CD8^+^ T effector and NK cells (2). Initial endeavors to procure IL2 from natural sources as a treatment for patients with cancer were disappointing. However, subsequent production via recombinant DNA technologies led to the development of recombinant human IL2 (aldesleukin) making it available for clinical investigation (3). Multiple clinical studies of aldesleukin treatment in renal cell carcinoma (RCC) and metastatic melanoma demonstrated compelling efficacy with durable responses and led to FDA approval for those indications in 1992 and 1998, respectively (4). High-dose IL2 (HD-IL2), in the form of aldesleukin, was among the first approved immuno-oncology (I-O) agents, preceding coining of the term.

Over the years, HD-IL2 use remained limited because of its severe toxicity profile stemming from induction of vascular leak syndrome (VLS), especially with the high-dose regimen. Hence, administration required dedicated care units such as the specialty team developed at the NCI Surgery Branch (5) that employed special training of medical personnel to manage VLS and other expected toxicities. The limited clinical use and lack of molecular profiling techniques at the time did not allow for correlating molecular profiles with clinical outcomes. Thus, the research opportunities to understand critical mechanisms of action in patients responding to treatment and identification of response predictors that could aid in patient selection and stratification may have been missed (6).

After the discovery and use of HD-IL2 in the rapidly evolving era of I-O, a dramatic increase in the understanding of the tumor and immune microenvironment has occurred. These insights have led to the development of a plethora of therapeutic approaches for clinical use, such as modulators of T-cell response including checkpoint inhibitory antibodies, T-cell receptor engager and/or stimulatory antibodies, suppressors of regulatory T cells (Treg) and myeloid-derived suppressor cells (MDSC), as well as adoptive cell therapies, oncolytic viruses, vaccines, Toll-like receptor agonists, and many more (for a review describing both antitumor mechanisms mediated by the immune system, and potential combination therapies, see Chen and colleagues (7), and Marshall and colleagues (8). While modulation of PD-1 and CTLA-4 with immune checkpoint inhibitors (ICI) has shown responses in up to 60% of the patients, a need remains to better understand the mechanisms driving resistance to these blocking antibody agents. In this context, the importance of modifying the tumor microenvironment is an area of substantial investigation. A common theme is the search for combination agents to elicit T- and NK-cell trafficking into tumors, stimulate proliferation of these immune cells, bolster their effector activity and survival, and enhance antigen presentation through induction of IFN responses, as elegantly presented by Chen and Mellman in the cancer-immunity cycle concept (7).

Given the known clinical efficacy of HD-IL2, this lymphokine has emerged as an agent that offers the potential for becoming a backbone I-O therapy. Hence, multiple next-generation IL2 drugs are moving through clinical trials as single agents or in combination with checkpoint inhibitors and other targeted I-O agents (9). Examples include (i) Nektar Pharmaceutical's NKTR-214, a form of IL2 reversibly conjugated with multiple polyethylene glycol (PEG) chains for slow release (10); (ii) Roche fusion IL2 variants including their PD-1 mAb/IL2v, RO6874281 (FAP mAb/IL2v), and cergutuzumab amunaleukin (CEA-IL2v) (11); (iii) Alkermes’ ALKS-4230, a fusion of IL2 to the extracellular domain of the alpha chain of its receptor (12); and (iv) Synthrox's/Sanofi's THOR-707 (SAR’245), an IL2 that leverages a synthetic biology platform for targeted PEG bioconjugation at a specific position in IL2 to prevent alpha chain engagement (13). Several additional constructs, many consisting of fusions to modules that target additional modulators of immune cell activity or cancer cell epitopes, are also in preclinical and/or clinical development.

This renewed interest in IL2 therapeutic potential occurs during a new era with more precise readouts for pharmacodynamic responses in the peripheral and tumor compartments. These include multicolor flow cytometry and molecular profiling methods which allow for peripheral lymphocyte phenotyping and gene expression analysis including enumeration of T-cell clones trafficking and expanding at the tumor site (14, 15). Of note, none of these technologies were available when HD-IL2 was first developed.

Previously, we studied patients with metastatic melanoma and mRCC treated with HD-IL2 and undertook sequential immune monitoring using flow cytometry, which revealed changes in immune profiles that were related to IL2 response (16). In the current study, we report on the pretreatment tumor molecular analysis, including RNA-sequencing (RNA-seq), combined with clinical annotation from a single-institution retrospective cohort of patients with metastatic RCC who underwent treatment as part of the dedicated HD-IL2 clinical program at Carolinas Medical Center (Atrium Health)/Levine Cancer Institute (LCI) between 2009 and 2013 (17). Patients in the current study had up to 17 years posttreatment follow-up. This cohort was compared with a publicly available cohort of patients with RCC treated with anti-programmed cell death-1 receptor (anti-PD-1) therapy who also underwent RNA-seq analysis (18). Genomic characteristics common to IL2 and anti-PD-(L)1 therapy response, as well as unique to each, are presented herein along with insight into rational combination strategies for these I-O agents.

## Materials and Methods

### Patient Samples

Patients were retrospectively identified as having mRCC and received treatment with HD-IL2 (aldesleukin) therapy within the LCI/Atrium Health hospital system (Charlotte, NC) between December 2009 and August 2013. Eligible patients had an available archived pretreatment formalin-fixed paraffin embedded (FFPE) tumor tissue sample from a primary or metastatic site with sufficient material from which to extract RNA and DNA. A total of 36 patients met the study criteria.

### Clinical Annotation

Demographic and clinical variables were collected from medical records and entered into a dedicated auditable database (REDCap; www.project-redcap.org) designed around a predefined data dictionary. Data entry and subsequent quality control (QC) review were performed by separate individuals. As appropriate, clinical variables were recorded at the time of initiation of IL2 therapy. HD-IL2 was given as a bolus intravenous injection (720,000 IU/kg) every 8 hours up to 14 doses followed by a 9- to 14-day rest period and then another week of systemic HD-IL2 (up to 14 doses; ref. 19). Overall survival (OS) was defined as the interval from IL2 treatment initiation to patient death. The Social Security Death Index was consulted whenever possible if death date was not available. Progression-free survival (PFS) from IL2 treatment was defined as the interval between initiation of initial IL2 treatment and disease progression, or the date of death in the absence of noted disease progression. In cases where a patient was still alive or the date of death was unknown, date of last contact was used in place to estimate the censored OS/PFS. Data regarding response to therapy were collected from medical records which included investigator and/or treating physician interpretation. Clinical benefit was defined as complete response (CR), partial response (PR), or stable disease (SD) for ≥6 months. Clinical response was defined as CR or PR. A total of 35 of 36 patients were evaluable for clinical benefit or clinical response. Baseline patient demographics and treatment responses are presented in Tables 1 and 2, respectively.

**TABLE 1:** Demographics of the study population.

Demographic	Pretreatment biopsy (*n* = 36)
Age, years, median (range)	64 (45–83)
Male sex, *n* (%)	25 (69%)
Race, *n* (%) White Black	33 (92%) 3 (8%)
Renal cancer type, *n* (%) Clear cell Papillary Other	30 (83%) 3 (8.5%) 3 (8.5%)
T, *n* (%) T1 T2 T3 NA	3 (10%) 5 (16%) 23 (74%) 5 (14%)
N, *n* (%) N0 N1 N2 Nx NA	9 (35%) 4 (29%) 1 (3%) 14 (39%) 8 (22%)
ECOG performance status, *n* (%) 0 1	33 (92%) 3 (8%)
Source of tumor for analysis, *n* (%)^a^ Primary tumor Biopsy of metastasis Mix of primary and metastatic tumor sample	23 (64%) 7 (19%) 6 (17%)
Time interval between tumor biopsy and IL2 treatment start, days, median (range)	95 (18–1803)
IL2 therapy line of treatment, *n* (%) 1st 2nd 3rd	32 (89%) 2 (6%) 2 (6%)
Number of course of IL2, *n* (%) 1 2 3 4	29 (81%) 3 (8%) 3 (8%) 1 (3%)
IL2 treatment duration, days, median (range)	24 (2–199)

^a^Adrenal gland biopsy is considered a metastasis.

**TABLE 2:** Treatment outcomes.

Outcomes	Pretreatment biopsy (*n* = 36)
Median duration of follow-up, mos (95% CI)	62.4 (52.9–NR)
Best response, *n* (%) CR PR SD PD NA **ORR**	1 (3%) 12 (34%) 6 (17%) 16 (46%) 1 37%
Clinical benefit^a^, *n* (%) Yes	15 (43%)
Median PFS^b^, mos (95% CI)	2.2 (1.9–17.8)
PFS at 6 months, (%) PFS at 1 year, (%)	43% 34%
Median OS^c^, mos (95% CI)	67.6 (31.6–NR)
OS at 6 months, *n* (%) OS at 1 year, *n* (%)	97% 88%

Abbreviations: mos, months; NR, not reached.

^a^Clinical benefit defined as CR+PR+SD for ≥6 months.

^b^PFS defined as time from IL2 treatment initiation to progression or death and was also the duration of response.

^c^OS defined as time from IL2 treatment initiation to death.

### Institutional Review Board Approval

Patient samples and corresponding clinical data were collected under an Institutional Review Board–approved protocol (LCI) that allowed for the waiver of informed consent for combined analysis of molecular data and relevant clinical and demographic data, provided that necessary public health information was removed, and dates were shifted prior to data transfer and subsequent analysis. Furthermore, the study was conducted in accordance with the Declaration of Helsinki.

### RNA-seq

Hematoxylin and eosin–stained FFPE sections underwent microscopic QC review by an anatomic pathologist to confirm histology diagnosis, evaluate percent tumor nuclei (≥5% required), percent necrosis and cellularity prior to macrodissection and dual DNA/RNA extraction using the truXTRAC FFPE total nucleic acid kit (Covaris). RNA quantitation was performed by Qubit measurement using ribogreen staining. RNA was qualitatively assessed for integrity by Agilent TapeStation gel electrophoresis. RNA samples approved for analysis (required 200 ng by ribogreen quantitation and a TapeStation DV200 value ≥20%) underwent library preparation using the Agilent SureSelect XT RNA direct prep kit. A no template control and positive control sample (NA12878 FFPE RNA) were included in each run. Libraries were individually captured, reviewed for appropriate size using a Bioanalyzer or TapeStation trace, and quantified (KAPA library quantitation) prior to equal molar pooling. Sequencing was performed on an Illumina NovaSeq6000 sequencer using an S1 flow cell to generate approximately 50M, 2 × 100 bp paired end reads. RNA-seq data were qualified and analyzed against other datasets within GeneCentric's archive. RNA-seq was successfully performed on 35 of 36 patients.

### RNA Expression Analyses

Expression values for the samples were derived from raw RNA-seq fastq files. Reads were aligned with STAR-aligner (GrCH38 ver. 22) to human assembly using the STAR/Salmon pipeline (20). Expression was quantified using the RSEM package (21) and the GrCH38 human transcriptome reference. Genes were filtered for a minimum expression count (at least 10 reads in at least five samples), and for a protein coding annotation by Ensemble (final set of genes = 16,901). Differential expression was assessed using the DESeq2 package (22) on this filtered set of genes. For all other analyses, all expression values were log_2_(1+*x*) transformed, median centered and upper quartile scaled.

### Analysis of Miao RCC Dataset

A separate dataset of whole transcriptome gene expression and clinical response data from a separate cohort of patients with RCC treated with the anti-PD-1 inhibitor nivolumab was accessed as Supplementary Data from Miao and colleagues (18) and comparted to the current HD-IL2 cohort.

### Gene Signatures

#### IL2 Treatment Response Nearest Centroid Classifier

A *de novo* 40-gene nearest centroid classifier was developed as detailed in Dabney and colleagues (23) for IL2 treatment response (e.g., CR/PR vs. SD/PD) using the 35 patients in the current study with clinical response data and corresponding tumor RNA-seq expression profiles. The genes comprising the classifier are included in Supplementary Table S1.

#### Tumor Microenvironment Immune Activation

Investigation of immune differences by objective response category, used previously published [Charoentong and colleagues (24), Bindea and colleagues (25)] and in-house derived immune marker signatures (26) that were calculated as average expression value of all genes in each signature gene set. Gene expression signatures included: Activated_B_cell, Activated_CD4_T_cell, Activated_CD8_T_cell, Activated_dendritic_cell, CD56bright_natural_killer_cell, CD56dim_natural_killer_cell, Central_memory_CD4_T_cell, Central_memory_CD8_T_cell, Effector_memory_CD4_T_cell, Effector_memory_CD8_T_cell, Eosinophil, Gamma_delta_T_cell, Immature_B_cell, Immature_dendritic_cell, Macrophage, Mast_cell, MDSC, Memory_B_cell, Monocyte, Natural_killer_cell, Natural_killer_T_cell, Neutrophil, Plasmacy-toid_dendritic_cell, Regulatory_T_cell, T_follicular_helper_cell, Type_1_T_helper_cell, Type_17_T_helper_cell, Type_2_T_helper_cell, IFNγ, and expression signatures based on 40 individual genes: Cd274(PD-L1), Pdcd1(PD-1), Pdcd1lg2(PD-L2), Ctla4, IL2ra, IL2rb, IL2rg, Cxcl9, Cxcl10, Ifi27, Ifit1, Ifit2, Ifit3, Mx1, Mx2, Oas2, Stat1, Stat2, Stat3, Stat4, Stat5a(STAT5), Tbx21, Itga1(CD49A), Itgae(CD103), Cd28, Tnfrsf9(4-1BB), Cd40, Nfat5(NFAT), type I IFNs (Ifna14, Ifna13, Ifna6, Ifna7, Ifna5, Ifna4, Ifna1, Ifna2, Ifna16, Ifnab1, Ifnk, Il6).

The 34-gene ccA/ccB RCC classifier, which was mainly developed in the localized setting to predict disease recurrence, was developed based on Brooks and colleagues (27) using calls on *n* = 380 The Cancer Genome Atlas (TCGA) kidney renal clear cell carcinoma (KIRC) data and a 34-gene list. Training was performed on two of three of the samples and testing on the remaining one of three. Centroids were directly fitted using the centered training set and all available genes.

The 32-gene list from the signatures based on T-effector/IFNγ response (Teff) and myeloid inflammation (Myeloid) reported in the IMmotion150 phase II trial of patients with mRCC treated with atezolizumab and bevacizumab versus atezolizumab or sunitinib (28) was evaluated in the current study. However, clinical data from the trial were not analyzed.

The signatures and individual genes are presented as heatmaps based on values generated using log_2_ median-centered expression values of genes making up different immune signatures and individual genes. Boxplots showing individual immune activation signatures or individual gene expression levels were also created and pairwise comparisons were conducted with *P* values displayed when Wilcoxon rank-sum test *P* values were < 0.05. Heatmaps and box plots were generated using R program version 3.5.3. Box plots show lower quartile, median and upper quartile expression data. Plot whiskers show the full distribution of the expression data.

### Statistical Analysis

OS and PFS analyses were conducted using Cox proportional hazards model with right censored endpoints. Associations between response to treatment and genomic markers were investigated using the Kruskal–Wallis test and Fisher exact test for quantitative and qualitative markers. Association between response to treatment and clinical characteristics were evaluated using Fisher exact test. Multivariable logistic regression models were used to test whether molecular subtype predicts response to treatment when adjusting for various genomic markers. All statistical analyses were conducted using R 3.6 software (http://cran.R-project.org)

Associations between categorical and continuous variables were evaluated using a *t* test or ANOVA depending on the number of categorical variable levels.

### Data Availability Statement

The raw RNA-seq data for this study were generated at OmniSeq and were used to generate the IL2 treatment response nearest centroid classifier, which is subject of a pending patent application. Therefore, the raw RNA-seq data are not publicly available, but are available upon reasonable request from the corresponding author.

## Results

### Responders to Treatment have High Immune Gene Signatures

**FIGURE 1:**
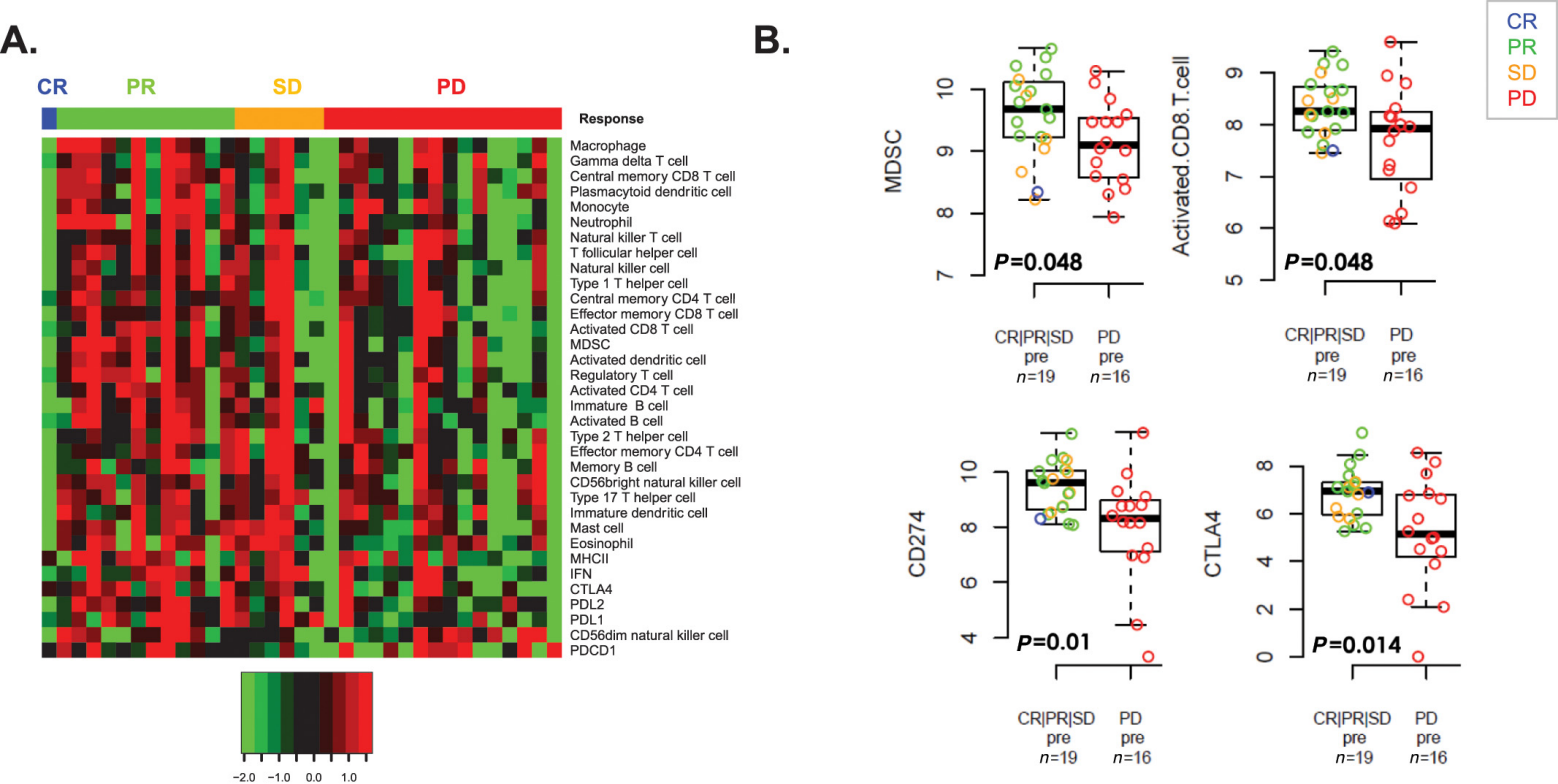
Immune signatures and markers are increased in patients responding to IL2 therapy compared with nonresponders. **A,** Heatmap showing expression of genes and immune gene signatures (rows) in IL2 pretreatment tumors (columns). Values are log_2_ median-centered expression values. Sample annotation bar represents clinical response. **B,** Boxplots of selected genes and immune signatures in pretreatment tumor samples with open circles colored by clinical response.

Since HD-IL2 is an immunotherapy and subsets of patients with RCC are responsive to IL2 and other I-O agents such as anti-programmed cell death-1 receptor or ligand (anti-PD-(L)1) antibodies, a select panel of immune gene signatures and individual genes were evaluated in the HD-IL2 pretreatment tumor specimens. Most tumors associated with clinical benefit (CR, PR, SD) or clinical response (CR, PR) had significantly higher expression levels of most signatures and individual genes (Fig. 1 and 4B), suggesting high immune infiltration in responsive tumors. The elevated expression levels of immune signatures in responsive tumors included several associated with immune suppression such as MDSCs (*P* = 0.01), neutrophils (*P* = 0.01), and Tregs (*P* = 0.02). Individual markers such as CD274 (PD-L1) (*P* = 0.04) and CTLA4 (*P* = 0.01) as well as signatures associated with effector cell types such as activated CD8 T cells (*P* = 0.05) and NK cells (*P* = 0.05) were also significantly elevated in responders based upon pretreatment tumor samples.

### A *de Novo* Gene Expression Classifier for HD-IL2 Response is Overrepresented by Immune-associated Gene Sets

To assess the immune signature findings in an orthogonal way and initiate the process of prioritizing genes that could support the identification of patients poised to respond to IL2, a gene expression classifier was derived. The classifier was trained based on clinical response (CR/PR vs. SD/PD) using a nearest centroid approach (23). Starting with 18,268 genes that were also available in TCGA, candidates were identified that were highly expressed and highly variable using cutoffs for the gene median (approximately the 25th percentile) and the gene variance (approximately the 70th percentile). This method resulted in the identification of 3,536 candidate genes for developing the response classifier. Here, the nearest centroid approach then ranks and selects genes according to CR/PR versus SD/PD t-statistics with the final number of genes determined by cross-validation agreement between response status and prediction.

**FIGURE 2:**
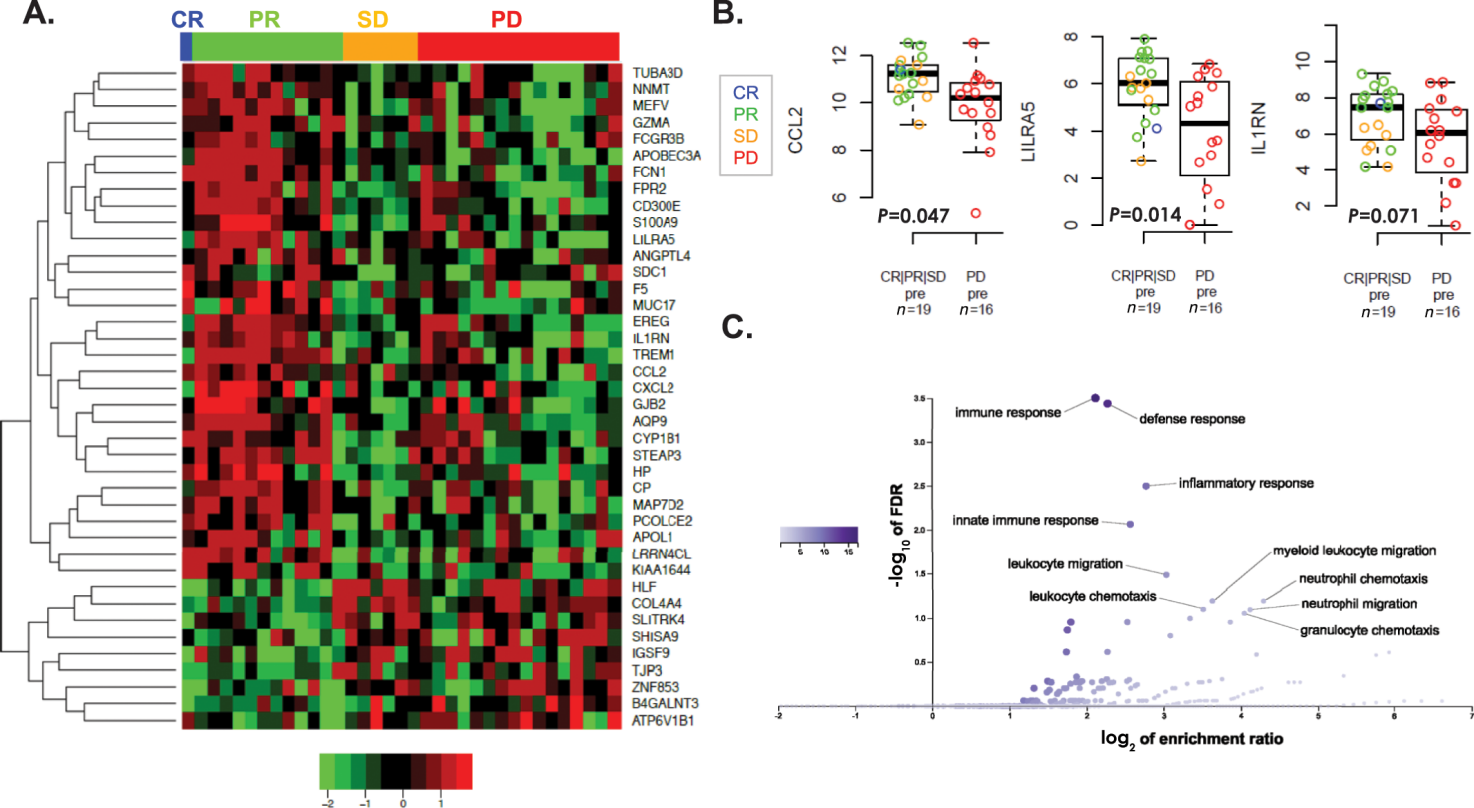
*De novo* gene expression classifier for IL2 clinical response. **A,** Heatmap showing expression of 40 genes (rows) in the classifier in IL2 pretreatment tumors (columns). Values are log_2_ median-centered expression values. Sample annotation bar represents clinical response. **B,** Boxplots of selected genes and immune signatures in pretreatment tumor samples with open circles colored by clinical response. **C,** Volcano plot of overrepresentation analysis of the 40 genes in the classifier and the associated gene sets.

Although the number of patients (*n* = 35 with evaluable clinical response data) limited the ability to divide the samples into training and test sets, the genes driving the classifier share similar immune associations of the observed immune gene signatures described above (Fig. 1). Figure 2A shows a heatmap of the classifier with many genes that are immune-associated being upregulated in samples associated with clinical response such as CXCL2, CD300E, LILRA5, CCL2, and GZMA (selected box plots; Fig. 2B). Overrepresentation analysis of the 40 genes in the classifier exclusively results in immune-associated gene sets (Fig. 2C). Hence, by an orthogonal approach selecting from over 3,500 genes, the immune gene signatures (Fig. 1) and the *de novo* IL2 response classifier (Fig. 2) suggest that patients demonstrating a clinical response to HD-IL2 have higher pretreatment levels of many immune-associated genes. And, although immune associated genes in the 40-gene classifier lack a clear association with a specific cell type, the classifier reflected gene ontologies such as “innate immune response” and “leukocyte migration” as well as immune cell types reflected in the immune gene signatures such as NK cells, neutrophils, and CD8^+^ T cells (Supplementary Table S2).

**FIGURE 3:**
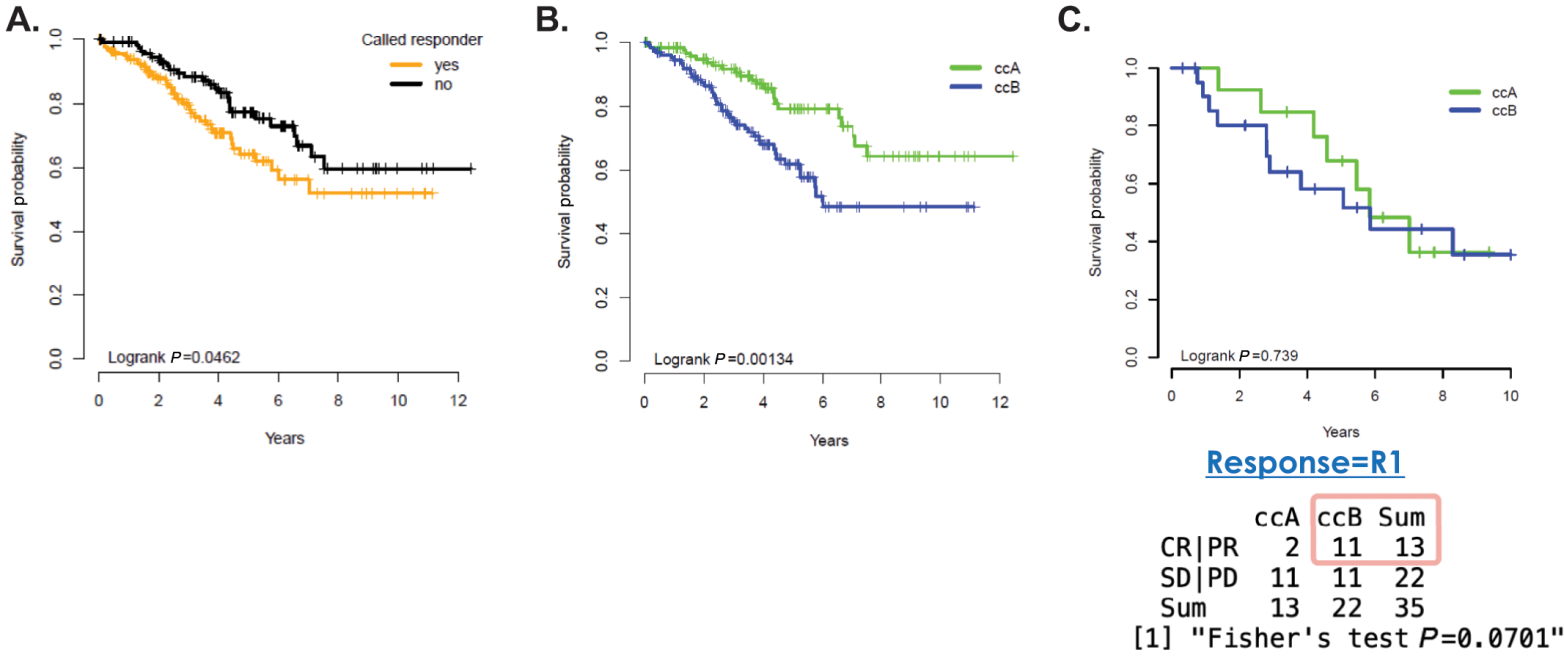
Worse OS in RCC based on higher immune classification methods in general but not in HD-IL2–treated patients. **A,** Kaplan–Meier plot of TCGA (KIRC, *n* = 253) tumor samples based on the *de novo* 40-gene IL2 response classifier. “Yes” indicates tumor samples that are classified with the patients in the IL2 cohort that have high immune infiltration and better clinical response. **B,** Kaplan–Meier plot of TCGA (KIRC, *n* = 253) tumor samples based on a canonical intrinsic subtype of RCC (the immune high ccB). **C,** Kaplan–Meier plot of tumor samples from patients with RCC treated with HD-IL2 (*n* = 36) based on a canonical intrinsic subtype of RCC (the immune high ccB) along with clinical response comparison.

Historically, high immune-gene signatures notably fail to predict clinical favorable outcomes (29). Using molecular intrinsic subtypes, tumors classified with high lymphocyte and macrophage (subtype “ccB”) infiltration based on gene expression associate with worse OS (27, 30). Furthermore, the immune infiltration associated with poor prognosis is characteristically associated with immunosuppressive cell types such as those reflected by the gene signatures (e.g., neutrophils, macrophages, and B cells; refs. 31–34). To investigate whether the immunologic features in the *de novo* IL2 response classifier were shared with the established prognostically poor ccB subtype, the *de novo* IL2 response classifier and an established ccA/ccB classifier (27) were applied to a randomly sampled subset of patients with RCC (*n* = 253) from TCGA (patients who are treated with various standards of care including IL2; Fig. 3A). Consistent with worse OS in the immune-associated subtype ccB (*P* = 0.001; Fig. 3B), the patients classified as responder “yes” (i.e., immune high) by the *de novo* IL2 response classifier have worse OS (*P* = 0.05; Fig. 3A). However, when the ccA/ccB classifier is applied to the HD-IL2 treated patients with RCC from the current study, the ccB subtype patients no longer have a worse OS (*P* = 0.7; Fig. 3C); however, the small sample size may contribute to the closing of the survival curves for the HD-IL2 patients. Furthermore, most (11/13) of the patients with a clinical response (CR/PR) are within the ccB subtype (*P* = 0.07). Thus, these results could support the hypothesis that IL2 treatment of ccB-subtype patients could improve the poor prognosis of this subtype and ameliorate their clinical response and survival.

### Immune Checkpoint Responders have a Distinct Immunogenomic Profile From IL2 Responders

Higher pretreatment immune infiltration or immune markers such as PD-L1 are reported to be associated with response for several tumor types (35). However, for RCC, reports suggest a more nuanced pretreatment immunologic profile of responding tumors where certain combinations of signatures or cell types are either predictive of nonresponse or response (28, 33). Some of the same pretreatment markers (e.g., GZMA and CD274) observed in IL2 tumors of responders in this study are also reported in pretreatment samples of anti-PD-(L)1 responder tumors McDermott and colleagues (28) and Helmink and colleagues (36). However, markers such as CXCL2 are described to be predictive of poor response to anti-PD-(L)1.

**FIGURE 4:**
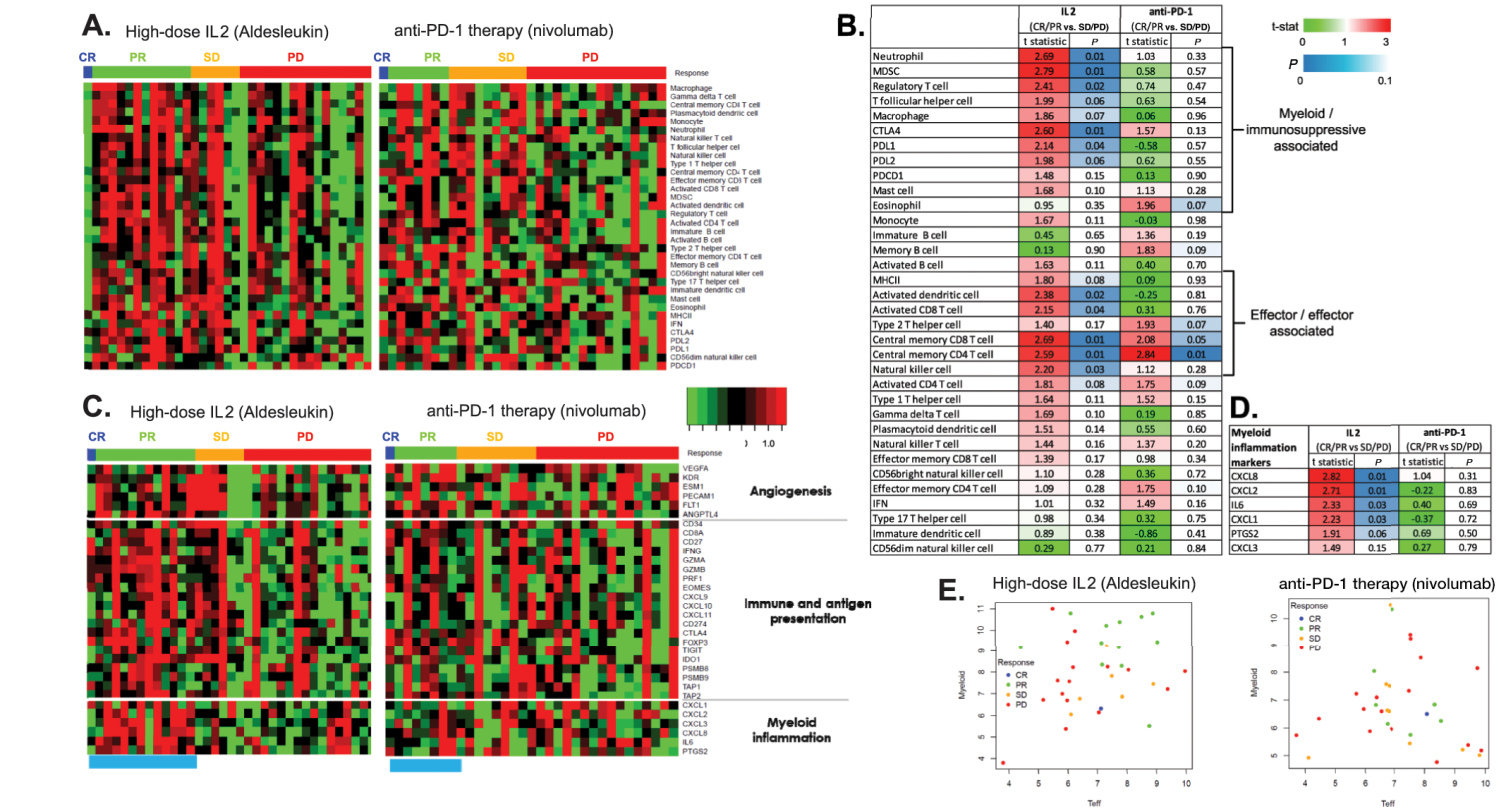
Comparison of immune cell and immune gene signatures (**A** and **B**), as well as myeloid inflammation and T-cell effector gene (**C**–**E**) expression profiles in patients with RCC treated with IL2 or anti-PD-1 therapy. **A,** Heatmaps showing expression of genes and immune gene signatures (rows) in IL2 pretreatment tumors (columns in left heatmap) and anti-PD-L1 (right heatmap). Values are log_2_ median-centered expression values. Sample annotation bar represents clinical response. **B,** Statistical results for the genes and immune gene signatures comparing pretreatment responsive tumors (CR+PR) to nonresponsive tumors (SD+PD) for IL2 and anti-PD-L1–treated patients. **C,** Heatmaps showing expression of genes (rows) in IL2 pretreatment (columns, left heatmap) and anti-PD-(L)1 (right) tumors. Values are log_2_ median-centered expression values. Sample annotation bar represents clinical response. Bright blue bars highlight the abundances of the myeloid inflammation genes for the responding tumors. **D,** Statistical results for the genes comparing pretreatment responsive tumors (CR+PR) to nonresponsive tumors (SD+PD) for IL2 and anti-PD-(L)1–treated patients. **E,** Scatter plot showing the relationship between the myeloid inflammation signature (Myeloid) and the T-cell effector (Teff) signature for IL2 and anti-PD-(L)1 pretreatment tumors, respectively.

To assess the possibility of immunogenomic distinctions between anti-PD-(L)1 responders and HD-IL2 responders, RNA-seq data from an anti-PD-(L)1–treated cohort (Miao cohort; with a similar distribution of response to the HD-IL2 cohort), were compared with the HD-IL2 responders (Fig. 4). Figure 4A and B shows the immune gene signatures for both cohorts. Although relative immune gene signature scores across the response categories in the cohorts look similar, statistical analysis shows that the significance and t-statistic values are distinct. As expected, both anti-PD1 and IL2 responders share some effector cell signatures such as specific CD4 and Th cell signatures (i.e., central memory CD4 T cell and type 1 Th cell signatures, respectively). In contrast, distinct from the anti-PDL1 cohort, signatures associated with immunosuppressive cell types are among the most significantly elevated in the HD-IL2 responders.

These distinctions in gene signatures associated with immunosuppressive cell types could be relevant to the choice of immunotherapy in RCC because similar immunosuppressive signatures have been shown to be associated with poor response to anti-PD-(L)1 therapy. Gene signatures based on T-effector/IFNγ response (Teff) and Myeloid were evaluated in the IMmotion150 phase II trial of patients with mRCC treated with atezolizumab and bevacizumab versus atezolizumab or sunitinib. The combination of Teff^HIGH^/Myeloid^LOW^ had strong PFS differences compared with patients with Teff^HIGH^/Myeloid^HIGH^ in the atezolizumab monotherapy arm [HR, 2.98; 95% confidence interval (CI), 1.68–5.29] (28). The less favorable response for Myeloid^HIGH^ patients is consistent with myeloid inflammation being implicated in suppression of the antitumor immune response (37).

To assess these anti-PD-(L)1 Myeloid signature results in the context of the immunosuppressive myeloid genes observed in the HD-IL2 cohort in this study, the relative expression levels of the genes reported in the IMmotion150 phase II trial (28) are presented in Fig. 4C–E for the pretreatment RCC specimens of both the HD-IL2 and the anti-PD1 cohort. For the anti-PD1 cohort, the profiles are similar to what is reported in IMmotion150 where the anti-PD-1–treated cohort responders have lower levels of genes associated with immunosuppression. In contrast, the HD-IL2 partial responders have higher levels of these genes (Fig. 4C). The Myeloid signature is comprised of six genes, with one of the genes, the myeloid chemokine CXCL2, also among the 40 genes in the *de novo* response HD-IL2 gene signature (Fig. 2). The six genes in the Myeloid classifier are mostly chemokines that play a central role in myeloid recruitment. Likewise, genes in the HD-IL2 *de novo* classifier such as CCL2, FPR2, L1RN, and S100A9 drive the gene set enrichments for factors such as myeloid leukocyte migration, neutrophil chemotaxis, and leukocyte chemotaxis (Fig. 2). Thus, in addition to the shared CXCL2 gene, other myeloid features are differentially present in anti-PD-(L)1 and IL2 responders.

## Discussion

HD-IL2 (aldesleukin), first approved in 1992, was the initial I-O therapeutic approach for RCC. This was followed by a targeted therapy era with multiple receptor tyrosine kinase inhibitors (RTKi) garnering FDA approval, including, sorafenib, sunitinib, pazopanib, axitinib, cabozantinib, lenvatinib, and tivozinib, as well as the mTOR inhibitors everolimus and temsirolimus. Resurgence of interest has occurred in I-O therapeutic options for RCC with development of ICIs. Anti-PD-(L)1–based therapy, approved for advanced RCC/mRCC, provides significant OS improvement over small-molecule targeted therapies when used as monotherapy or combination therapy. The combination of nivolumab plus ipilimumab approved after Checkmate 214 study showed improved OS compared with sunitinib, with updated 5-year OS data reported recently (38). For the intent-to-treat population, the OS was 48% for the combination compared with 37% for sunitinib (HR, 0.72; *P* < 0001). Several combinations of I-O plus RTKis, including pembrolizumab plus axitinib, avelumab plus axitinib, nivolumab plus cabozantinib, and pembrolizumab plus lenvatinib have shown impressive responses and improved relapse-free survival and have been FDA approved (39, 40). New “next-generation” IL2 agents are also under clinical investigation, including NKTR-214 (10), THOR-707/SAR’245 (NCT04009681), ALKS-4230 (12), RO6874281 (11), which are designed for improved selectivity and tolerability, while maintaining or improving upon the antitumor activity of HD-IL2.

Even with survival improvements from new anti-PD-(L)1 therapeutic agents, a significant proportion of patients do not respond to these treatments. For example, almost half of the patients treated with pembrolizumab + axitinib or avelumab + axitinib in the first-line setting (41, 42) and three quarters of patients treated with nivolumab in the second-line or greater setting lack a treatment response (43). However, again, more than half of patients with RCC fail to achieve an objective clinical response even with the combination of anti-PD-1 and anti-CTLA4 therapy (44). Thus, alternative I-O combination regimens merit investigation, including anti-PD-(L)1 in combination with “next-generation” IL2, as supported by preclinical results and by ongoing clinical trials (13, 45, 46).

With multiple I-O treatment modalities now either in clinical development or approved for clinical use, a need exists for new genomic expression and transcriptomic response markers that permit a precision medicine approach to selecting optimal therapeutic regimens for individual patients, particularly considering that traditional IHC tests such as PD-L1 are not always predictive of response (47, 48). Predictive genomic biomarkers may aid in selecting patients most likely to respond to I-O treatments, whether they are markers of response to I-O therapy in general or to one treatment option over another [e.g., anti-PD-(L)1 vs. IL2]. Furthermore, the explosion of IL2 therapeutic constructs in the clinic necessitates better tools to evaluate and predict responses for assessment of potential best-in-class agents. As a baseline for assessing next-generation IL2 constructs, we evaluated the clinical and genomic characteristics of a well-defined cohort of patients with mRCC treated with HD-IL2 (aldesleukin) and compared the findings with previously published cohort of patients with mRCC treated with anti-PD-1 therapy (nivolumab). Prior to our analysis, only two publications were publicly available, entailing extremely limited sample numbers and analytic tools (6, 49). Both of the cohorts we analyzed, demonstrated a similar percentage of patients with stable disease or better clinical responses with treatment (54% HD-IL2 and 57% anti-PD-L-1). Also, similar to what has been previously demonstrated for I-O therapy in general, patients demonstrating a treatment response generally had increased overall expression of immune cell and gene signatures in their pretreatment tumors (28, 36).

There are potential limitations of the current study related to it being a retrospective cohort that reflects real-world HD-IL2 in a single dedicated treatment unit. Namely, the cohort is a relatively small sample size and while most of the patients had a clear-cell pathology, a small minority had other non–clear-cell pathology. Also, patients were relatively healthy (e.g., majority being ECOG 0 and treatment naïve) and RECIST were not uniformly used to record treatment response. Despite these potential limitations, the cohort and findings associated with its analysis provide insight into potential predictive markers of HD-IL2 response, and overall tumor biology. These insights could be utilized in the clinical development of novel next generation IL2 agents and place those agents in context with other I-O therapies such as anti-PD-(L)1. For example, an objective of this retrospective analysis was to identify potential genomic differences related to clinical response to I-O therapies with distinct mechanisms of action [e.g., anti-PD-(L)1 vs. HD-IL2]. When dissecting the gene expression differences by immune cell and/or gene signature, both HD-IL2–treated and anti-PD-1–treated responders show similar increased expression of effector and effector-associated cell/gene signatures compared with nonresponders. In contrast, myeloid and immunosuppressive-associated cell /gene signatures were among the most elevated in the patients demonstrating a response to HD-IL2, but this was not observed with anti-PD-1 treatment. Expression of targets for both anti-PD-(L)1 and CTLA4 therapy (e.g., CD274 and CTLA4) was also increased in patients who demonstrated clinical responsiveness to HD-IL2 therapy, but this was not observed in the anti-PD-1–treated patients. The underlying explanation for these differences observed in HD-IL2 versus anti-PD-1 is a matter for speculation. Some of the biomarker differences could be interpreted as pharmacodynamic evidence of bioactivity of these medicines, such as an increase in Tregs stimulated by HD-IL2 (aldesleukin). Other biomarker signatures with differential significance were present prior to treatment, suggesting a predilection for one I-O therapy over the other based on patient baseline tumor microenvironment characteristics. An example is found in the unique association of myeloid and/or suppressor signatures with HD-IL2 responders, which potentially could be exploited to select patients that are ideally suited for treatment with IL2 or combination of IL2 and anti-PD-(L)1. Recent updated results from phase I/II investigation of IL2 (NKTR-214) + anti-PD-1 (nivolumab) in patients with metastatic melanoma demonstrated the utility of pretreatment tumor biomarkers as potential predictors of response with increased CD8^+^ tumor-infiltrating lymphocytes and IFNγ gene expression associated with higher objective response rate (50). The current study also demonstrated that increased CD8 T-cell expression (activated CD8 T-cell signature; Fig. 4) was increased in patients with mRCC who responded to HD-IL2 compared with nonresponders, however this was not observed with anti-PD-1 treatment.

Several reports indicate that aspects of the elevated immune markers observed for HD-IL2 responders predict mRCC anti-PD-(L)1 response such as B cells, dendritic cells, etc. But, in contrast to the widespread elevation of nearly all immune markers observed in pretreatment HD-IL2 responders, several reports indicate that distinct combinations of immune markers (including downregulation) predict response to anti-PD-(L)1 treatment including recent results with atezolizumab which convincingly showed a survival benefit for patients with a Teff^HIGH^/Myeloid^LOW^ gene signature compared with those with a Teff^HIGH^/Myeloid^HIGH^ (28).

Nonclinical evaluations of I-O treatments in relevant animal models support the retrospective clinical analyses described above with a similar differential responsiveness demonstrated between IL2 and anti-PD-(L)1 treatment. For example, using the syngeneic CT-26 colorectal cancer model, the next generation not-alpha IL2 agent THOR-707/SAR’245 resulted in a significant increase in PD-L1 and CTLA4 receptor gene expression when combined with anti-PD-1 therapy, with the latter also increased with single-agent THOR-707/SAR’245 treatment (13).

Also, as part of the search for potential genomic markers of clinical response to I-O therapies with distinct differences in mechanism of action, we developed a *de novo* gene classifier that could be used as a baseline for further evaluating genomic effects of novel next-generation IL2 agents. Overrepresentation analysis determined that the 40 genes in the HD-IL2 response classifier were primarily immune related. This classifier was subsequently evaluated in a larger TCGA cohort of patients with RCC undergoing a broad range of treatment modalities, revealing significant differences in survival between classifier positive and negative patients. However, the classifier needs to be further evaluated with other datasets retrospectively and validated prospectively, as well as undergo further evolution as datasets become available for patients treated with next-generation IL2 targeted agents.

In summary, this retrospective analysis of patients with mRCC demonstrated both shared and unique immune cell–related signatures and genes with regards to responsiveness to treatment with HD-IL2 or anti-PD-1 therapy. Those differences provide support for further investigation into identification of potential predictive response signatures that could better select patients who are best suited for a particular I-O therapy as a single-agent or combination therapy. The deeper understanding into the molecular mechanisms of action for these therapies may provide insight into ways they could synergize with each other. A molecular biomarker driven approach may not only improve patient survival and response rates, but it also could reduce the costs associated with trial-and-error approaches. A biomarker signature may also reveal patient-specific associations with toxicity that aid in steering patients toward better tolerated therapeutic options and thereby improve quality of life.

## Supplementary Material

Table S1 and Table S2 40 gene IL-2 response classifier and related immune cell types

Supp Table test this is a test
