# Supplementary material for: Distinct Predictive Immunogenomic Profiles of Response to Immune Checkpoint Inhibitors and IL2: A Real-world Evidence Study of Patients with Advanced Renal Cancer
Source: Cancer Res Commun. 2022 Aug 30;2(8):894–903. doi: 10.1158/2767-9764.CRC-21-0153 (PMC10010312; doi:10.1158/2767-9764.CRC-21-0153)
Supplement: Table S1 and Table S2 — 40 gene IL-2 response classifier and related immune cell types [file crc-21-0153-s01.docx]

**Supplemental Table 1. 40 genes included in the IL-2 treatment response classifier.**

| **Gene** | |
| --- | --- |
| S100A9 | LRRN4CL |
| IGSF9 | NNMT |
| FCGR3B | B4GALNT3 |
| F5 | GJB2 |
| SDC1 | AQP9 |
| CYP1B1 | MEFV |
| ATP6V1B1 | SHISA9 |
| IL1RN | HP |
| STEAP3 | CCL2 |
| TUBA3D | HLF |
| COL4A4 | CD300E |
| PCOLCE2 | TJP3 |
| CP | ANGPTL4 |
| CXCL2 | FPR2 |
| EREG | LILRA5 |
| GZMA | APOL1 |
| TREM1 | APOBEC3A |
| ZNF853 | KIAA1644 |
| MUC17 | MAP7D2 |
| FCN1 | SLITRK4 |

**Supplemental Table 2. Immune genes in 40 gene classifier- association with immune cell types and GO biological terms.**

| **Immune genes in 40 gene classifier** | | **Association with immune cell type and source** | | | **GO terms** | | | |
| --- | --- | --- | --- | --- | --- | --- | --- | --- |
|  |  | **Gene presence in immune gene signature from Figure 1A** | **DICE (https://dice-database.org/)** | **Human protein atlas  (https://** [**www.protein**](http://www.protein) **atlas.org/)** |  | | | |
| APOL1 | apolipoprotein L1 | NA | NK cells | NK/neutrophil | GO:0006955 | GO:0006952 | GO:0045087 |  |
| AQP9 | aquaporin 9 | Gamma delta T cell | monocyte | neutrophil | GO:0006955 |  |  |  |
| CCL2 | C-C motif chemokine ligand 2 | NA | CD8; monocyte | monocyte | GO:0006955 | GO:0006952 | GO:0045087 | GO:0006954 |
| CD300E | CD300e molecule | Effector memory CD4 T cell | monocyte | monocyte | GO:0006955 | GO:0006952 | GO:0045087 |  |
| CXCL2 | C-X-C motif chemokine ligand 2 | NA | monocyte | NK, other effectors | GO:0006955 | GO:0006952 | GO:0006954 | GO:0050900 |
| EREG | epiregulin | NA | monocyte | monocyte | GO:0006955 | GO:0006952 | GO:0045087 |  |
| FCGR3B | Fc fragment of IgG receptor IIIb | Central memory CD8 T cell | monocyte; NK | neutrophil | GO:0006955 |  |  |  |
| FCN1 | ficolin 1 | Mast cell | monocyte | monocyte | GO:0006955 | GO:0006952 | GO:0045087 |  |
| FPR2 | formyl peptide receptor 2 | Macrophage | monocyte | monocyte | GO:0006955 | GO:0006952 | GO:0006954 | GO:0050900 |
| GZMA | granzyme A | Activated CD8 T cell | NK, other effectors | Effector cell types | GO:0006955 |  |  |  |
| HP | haptoglobin | NA | monocyte | eosinophil; monocyte | GO:0006952 | GO:0006954 |  |  |
| IL1RN | interleukin 1 receptor antagonist | Central memory CD8 T cell | monocyte | neutrophil | GO:0006952 | GO:0006954 | GO:0050900 |  |
| LILRA5 | leukocyte immunoglobulin like receptor A5 | Immature dendritic cell | monocyte | monocyte | GO:0006952 | GO:0045087 | GO:0006954 |  |
| MEFV | MEFV, pyrin innate immunity regulator | NA | monocyte | neutrophil; monocyte | GO:0045087 | GO:0006954 |  |  |
| MUC17 | mucin 17, cell surface associated | NA | ND | ND | GO:0045087 |  |  |  |
| S100A9 | S100 calcium binding protein A9 | MDSC | monocyte | neutrophil; monocyte | GO:0045087 | GO:0006954 | GO:0050900 |  |
| SDC1 | syndecan 1 | NA | ND | B cell; plasmablast | GO:0006954 | GO:0050900 |  |  |
| TREM1 | triggering receptor expressed on myeloid cells 1 | Activated dendritic cell | monocyte | neutrophil | GO:0045087 | GO:0006954 | GO:0050900 |  |
| **Go terms represented:** | | NA = not applicable because gene is not represented in these immune gene signatures; ND = no data | | | | | | |
| GO:0006955 immune response | |  |  |  |  |  |  |  |
| GO:0006952 defense response | |  | | | | | | |
| GO:0045087 innate immune response | |  | | | | | | |
| GO:0006954 inflammatory response | |  | | | | | | |
| GO:0050900 leukocyte migration | |  | | | | | | |
